# Early-Stage Physical Therapy for a Patient With Proximal Tibial Fracture With Acute Compartment Syndrome and Neurovascular Deficits Managed With External Fixation Complicated by Chronic Osteomyelitis: A Case Report

**DOI:** 10.7759/cureus.31333

**Published:** 2022-11-10

**Authors:** Abhishek Daf, Avanti A Gachake, Palash R Satone, Om C Wadhokar, Pratik Phansopkar

**Affiliations:** 1 Physiotherapy, Ravi Nair Physiotherapy College, Datta Meghe Institute of Medical Sciences, Wardha, IND; 2 Musculoskeletal Physiotherapy, Ravi Nair Physiotherapy College, Datta Meghe Institute of Medical Sciences, Wardha, IND

**Keywords:** compartment syndrome, foot drop splint, sensory re-education, functional electrical stimulation, orthopedics & physical therapy, ilizarov ring fixator, foot drop, extra-articular proximal tibial fracture, compartment syndrome leg

## Abstract

Tibial fractures occur following low-energy and high-energy trauma resulting from indirect and direct impacts, respectively. High-velocity trauma like road traffic accidents usually results in open fractures of the tibia associated with acute compartment syndrome, posing a serious threat. Thus, this injury requires prompt operative management with fasciotomy followed by fixating the fracture with an external fixator to avoid infection. Despite all the efforts, sometimes this condition may lead to osteomyelitis of the tibia requiring further care. Meanwhile, the patient has to be kept immobilized for a longer duration of time, which allows further complications to occur. Therefore, structured physiotherapeutic management of patients postoperatively is of immense necessity to prevent complications associated with prolonged immobility and achieve an optimal level of functional independence, thereby facilitating the patient to come back to near-normal life as soon as possible. Our case report provides a structured early-stage postoperative physical therapy treatment protocol for a patient with a proximal tibial fracture with acute compartment syndrome and neurovascular deficit managed with Ilizarov fixation.

## Introduction

Tibial fractures are among the most common long bone fractures, which occur as a result of trauma. The fractures of the tibia occur following both low-velocity as well as high-velocity mechanisms that result from indirect and direct impacts, respectively. The low-velocity injuries lead to spiral fractures with minimal soft-tissue injuries, whereas the high-velocity injuries result in oblique fractures with substantial comminution and soft-tissue damage associated with acute compartment syndrome, bone infection, and adjacent bone fractures [1]. According to the AO/OTA (Arbeitsgemeinschaft für Osteosynthesefragen/Orthopaedic Trauma Association) classification, tibial fractures are classified as follows based on the segment of the bone involved: type 1 - proximal tibial fracture; type 2 - diaphyseal fracture; and type 3 - distal tibia fracture. The proximal tibial fractures involve the proximal metaphyseal region of the bone that may or may not extend into the adjacent knee joint. Based on this, proximal tibial fractures are further sub-classified under AO/OTA classification as type 1A (extra-articular), type 1B (partial intra-articular), and type 1C (complete intra-articular). They may also be open or closed fractures [2]. Open proximal tibial fractures occur in the majority of cases. The proximal segment of the tibia is more prone to open fracture and skin disintegration as its anterior and medial aspect lies directly below the skin due to the lack of muscle mass. The tibia's minimal soft tissue covering also limits the periosteal blood supply to the bone, which is critical for speedy healing [3].

Extra-articular proximal tibial fractures account for approximately 5-11% of all tibial shaft fractures that often result from violent trauma. They result in extensive injuries, which involve bone and adjacent soft tissues [4]. Tibial injuries require prompt management owing to their potentially lethal consequence of compartment syndrome, which occurs in 8.1% of tibial shaft fractures compared to 1.6% and 1.4% of proximal-third and distal-third tibial fractures, respectively. Compartment syndrome occurs when the intra-compartmental pressure increases, which disrupts blood supply and eventually results in function loss [5,6]. Clinical features of compartment syndrome include inflammation, pain, edema, hypoesthesia or paresthesia, stiffness, and muscular paralysis [7].

Tibial fractures can be treated either by conservative means or operative means. Conservatively managed tibial fractures are often associated with complications like the failure of union, malalignment, joint stiffness, and instability. Therefore, operative methods are preferred to manage these fractures. The operative approaches available are external fixation and internal fixation. The options for internal fixation are intramedullary nailing (IMN) and plating; the choices for external fixation are Ilizarov ring fixators [8]. The external fixation technique is one of the most commonly used operative methods for open tibia fractures but is usually associated with complications like pin tract infection and loosening of fixation, non-union, and malunion of the fracture. Therefore, surgeons consider internal fixation using IMN for open tibia fractures [9]. The initial treatment for the tibial fractures associated with acute compartment syndrome usually includes a fasciotomy procedure and external fixation followed by secondary management with internal fixators [6]. Fasciotomy within six hours of injury is an appropriate method to treat tibial fractures associated with compartment syndrome. Meanwhile, the fasciotomy procedure may not be preferred when tissue pressure remains elevated even after 36 hours, as it may have already led to irreversible tissue necrosis. Debridement is necessary in case the infection has occurred to prevent sepsis or other consequences [10]. Delayed complications may occur after initial treatment or in response to treatment. The incidence of chronic osteomyelitis (CO) is 15% and 350% after closed and open fractures, respectively. Initial medical management for CO includes debridement and intravenous antibiotics; although, in extreme situations that may lead to local or systemic spread of infection or skeletal fragility, care may entail surgical drainage, wound cleaning, stabilization, and soft tissue repair [11]. Here, we are reporting a case of a patient with a proximal tibial fracture with acute compartment syndrome and neurovascular deficit managed with fasciotomy and external fixation by a non-bridging fixator, which was then complicated with CO; thus, further, the fracture was fixated externally with an Ilizarov ring fixator. We present here the physiotherapeutic management of the given case.

## Case presentation

Patient details

A 32-year-old male patient, a truck driver by occupation with right-handed dominance, was admitted to the male orthopedic ward following an operative procedure on the right lower extremity. He complained of pain over the operated site, inability to move the right ankle joint, and loss of sensation over the right foot. The pain was present over the lateral aspect of the right leg and heel, rated 4/10 during left lower limb movements and transfers and 2/10 at rest on the numerical pain rating scale, dull aching in nature, aggravated during transfers and on moving the affected limb, and relieved on medications and rest with no diurnal variations. The patient gave no history of any co-pathology and consumption of regular drugs or medications.

The patient was all right three months back. Then he had an alleged history of a road traffic accident, sustaining an injury to the right lower limb. The patient was conscious after the incident. The bystanders soon rushed him to a hospital in Nagpur. At the hospital, a doctor examined him and advised a radiographic investigation. Based on the X-ray findings, he was diagnosed with a case of type 1A proximal tibia fracture (according to AO/OTA classification) with impending compartment syndrome with neurovascular deficits. The patient was then managed with an external fixator application and fasciotomy and admitted to the orthopedic ward for approximately six weeks. The patient was discharged with an external fixator still in place but soon started experiencing pain in the operated extremity, which was throbbing in nature and progressed gradually over the next few days. The pain was associated with swelling over the operated site, which increased over the next few days. Then, he noticed a discharge from the fasciotomy wound. Thus, the patient visited a private physician. The physician examined him, advised him of several investigations, and then diagnosed him with chronic osteomyelitis of the tibia. Then, he was referred to another hospital, for further management. The first operation was to remove the external fixator and clean the wound. Then, a cylindrical slab was applied. The second operation was a check dressing and wound debridement. The third operation was an Ilizarov ring fixation of the right lower limb using seven plane wires, olive wire, and three Ilizarov rings of 160 mm. Then the patient was referred to musculoskeletal physiotherapy for postoperative management on postoperative day four.

Clinical evaluation

Observation

**Figure 1:**
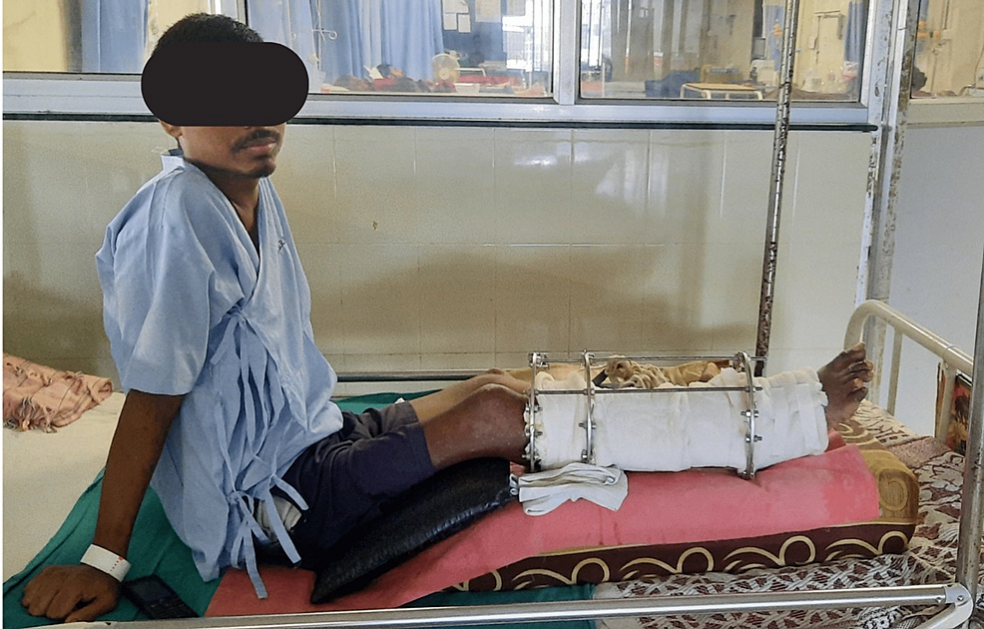
Patient in the long sitting position with affected extremity supported over the pillow.

**Figure 2:**
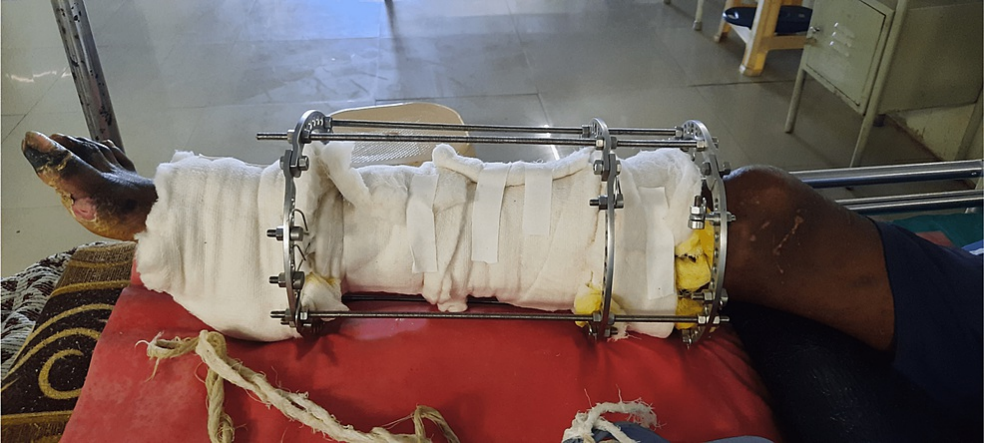
Ilizarov fixator applied with a slab over the right leg.

**Figure 3:**
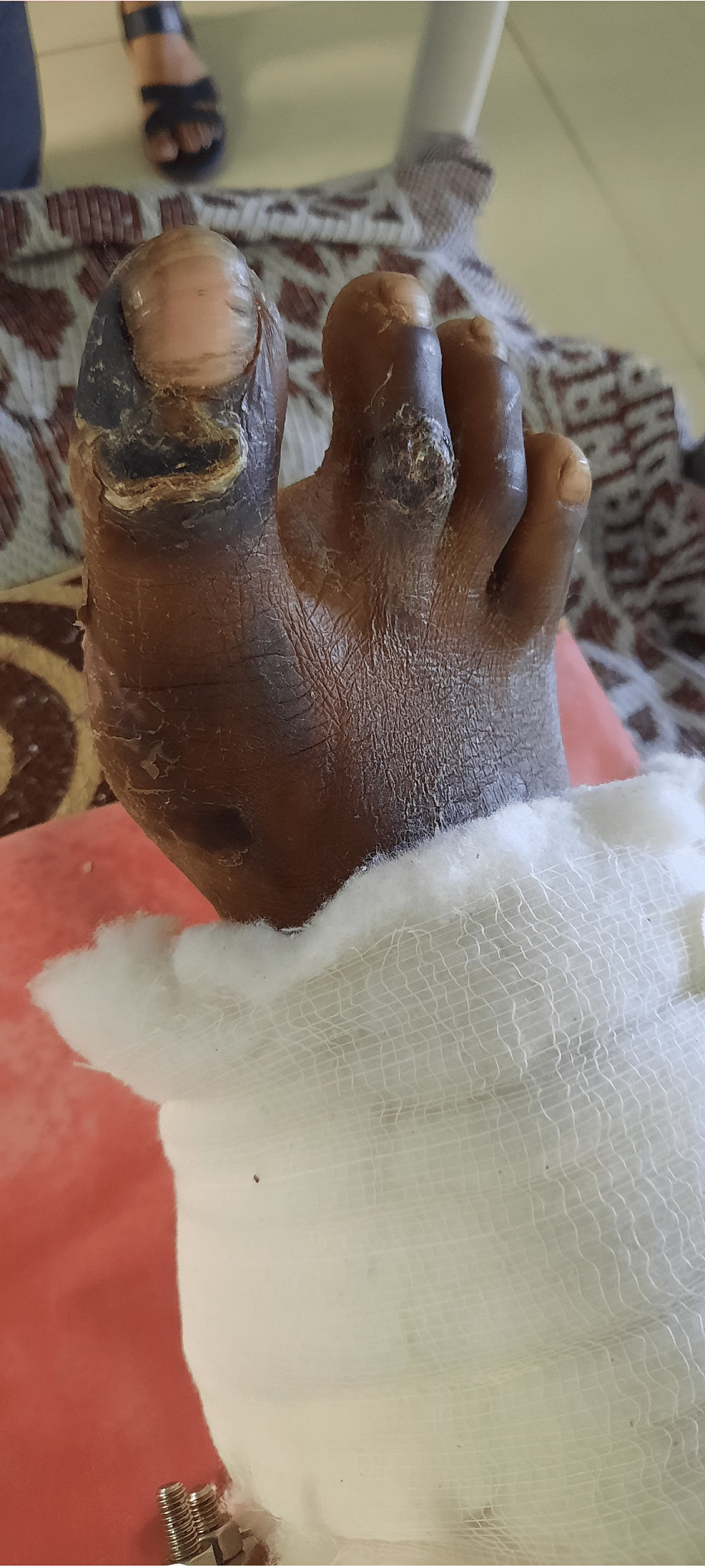
Blackish discoloration and dry skin over the dorsum of the right foot with wounds over the dorsum of the great toe and second toe of the right foot.

**Figure 4:**
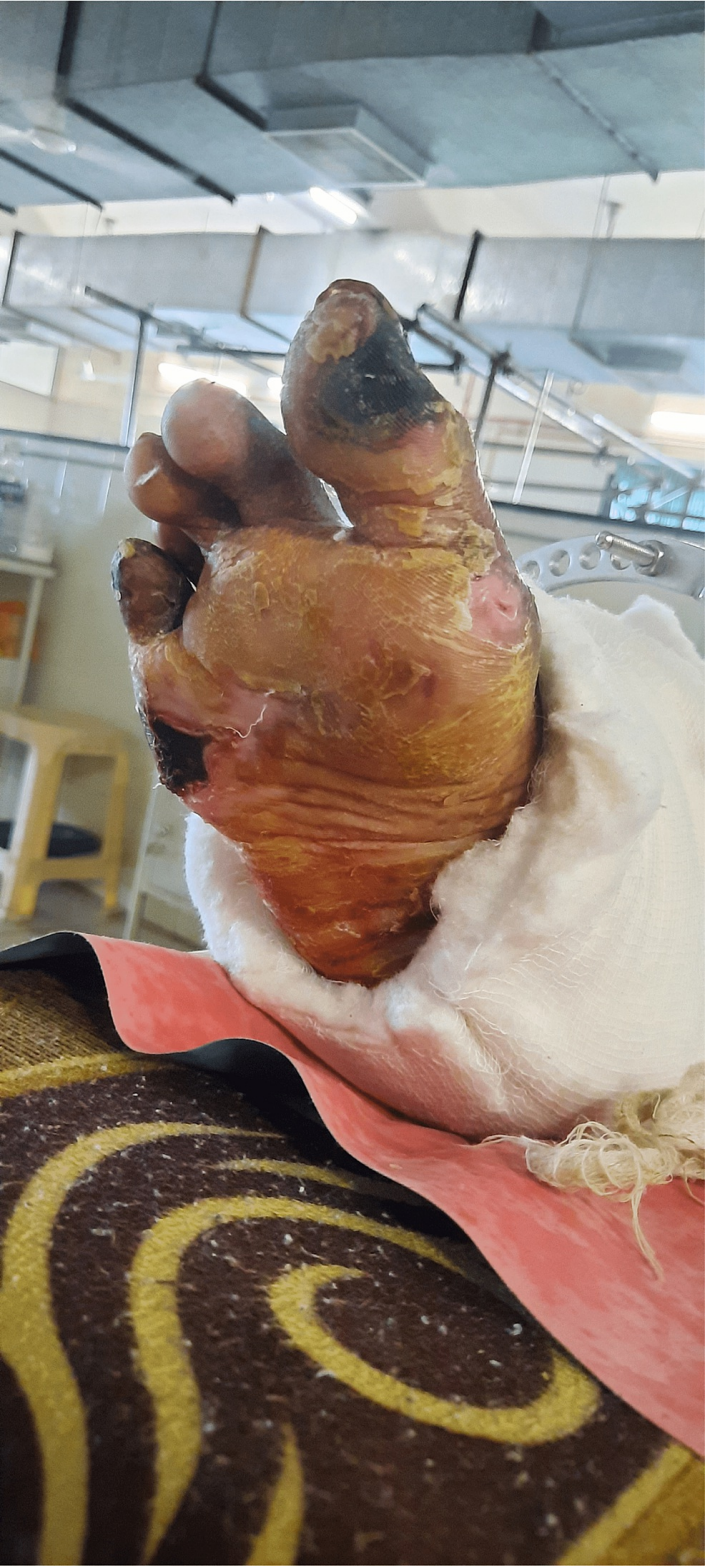
Drying and yellowish discoloration of the skin over the sole of the right foot with a wound over the plantar aspect of the great toe.

**Figure 5:**
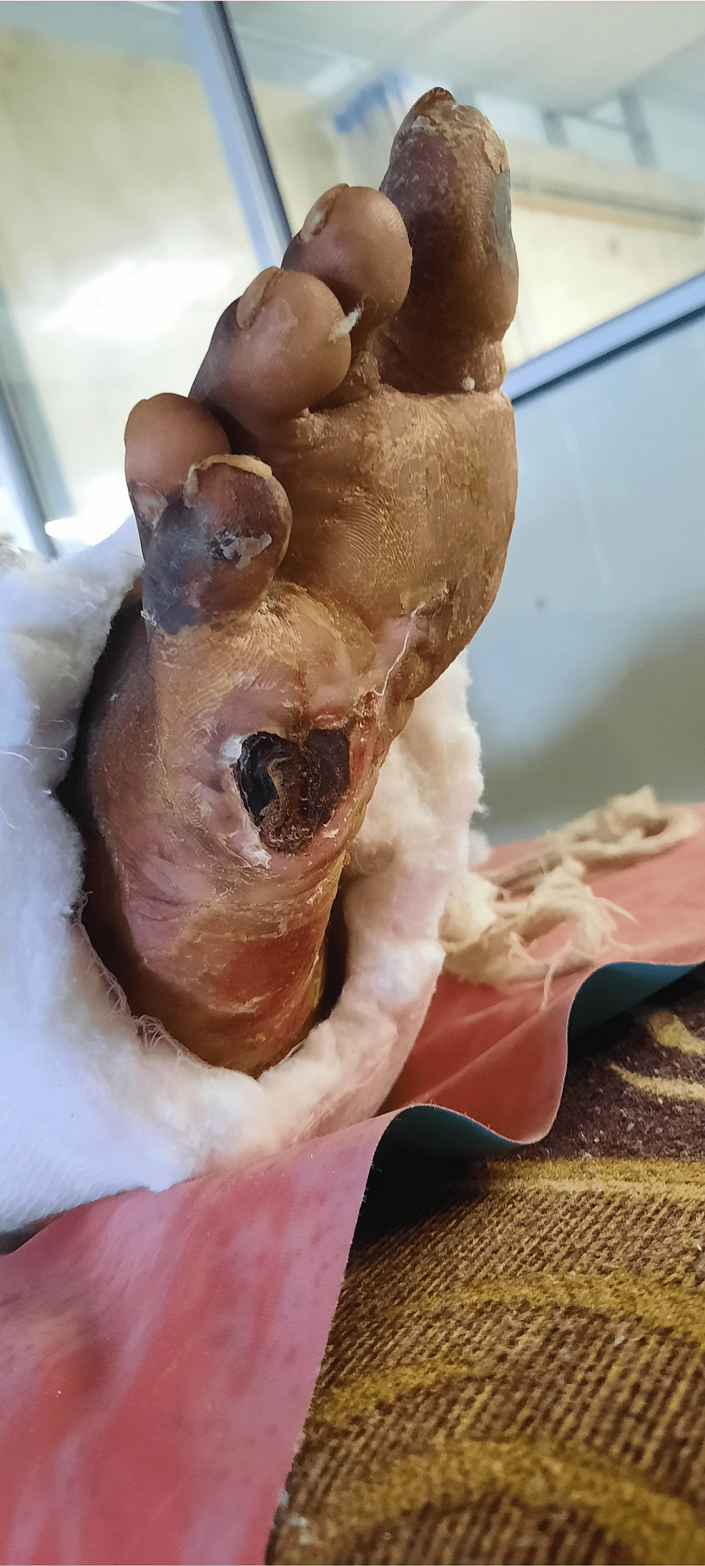
A wound over the sole of the right foot just below the fifth toe.

The patient was in a long sitting position with the operated extremity supported on a pillow with the hip and knee slightly flexed (approximately 30 degrees) and plantar flexion at the ankle, as shown in Figure 1. The Ilizarov ring fixator with the slab was present over the right leg, as shown in Figure 2. There was blackish discoloration and dry skin over the dorsum of the right foot with wounds over the dorsum of the great toe and second toe of the right foot, as shown in Figure 3. There was yellowish and dry skin over the sole of the right foot with wounds over the plantar aspect of the great toe and just below the fifth toe, as shown in Figures 4, 5. The swelling was present over the right knee and foot. There was also the wasting of the quadriceps femoris.

Palpation

Grade 1 tenderness over the lateral aspect of the leg and the lateral malleolus was present. Further, the spasm was present in the calf muscle. Grade 1 edema over the right foot was confirmed. We used the figure of eight method to measure the swelling in the right foot and obtained a reading of 34.5 cm on an inch tape. We performed a similar measurement on the left foot and found a value of 35 cm on inch tape. In comparison, reading on the right foot was 0.5 higher than that of the left foot. This confirmed the swelling of 0.5 cm over the right foot.

Examination

There was a restriction of range of motion (ROM) for the affected right lower extremity. The ROM was in normal ranges for the unaffected extremities. Strength grading for the right-side quadriceps femoris and hamstrings was weak and painful on resisted isometric muscle testing. On sensory examination, light touch and pinprick sensations were diminished over the dorsum and sole of the right foot. We measured the girth of the right and left thighs with inch tape at three levels (6 inches, 9 inches, and 12 inches) from the respective anterior superior iliac spine (ASIS). Then, we compared the values of both sides at each level, as shown in Table 1. In comparison, there was atrophy of the quadriceps femoris of the right lower extremity.

**Table 1:** Representation of comparison of values of the girth measurement of right and left thigh. The values of girth measurement for the right and left thighs were taken at levels 6 inches, 9 inches, and 12 inches from the anterior superior iliac spine (ASIS). The values on the right and left sides were compared and differences of 4 cm, 7 cm, and 6 cm were found at levels 6 inches, 9 inches, and 12 inches, respectively, with lower values on the right side. This provides an impression regarding the atrophy of the quadriceps femoris of the affected right lower limb due to prolonged immobilization and inactivity.

Thigh (measured at three levels from ASIS)	Right	Left	Difference
6 inches	39 cm	43 cm	4 cm
9 inches	33 cm	40 cm	7 cm
12 inches	29 cm	35 cm	6 cm
Significance: atrophy of quadriceps femoris of the right lower extremity is confirmed.			

Investigation

**Figure 6:**
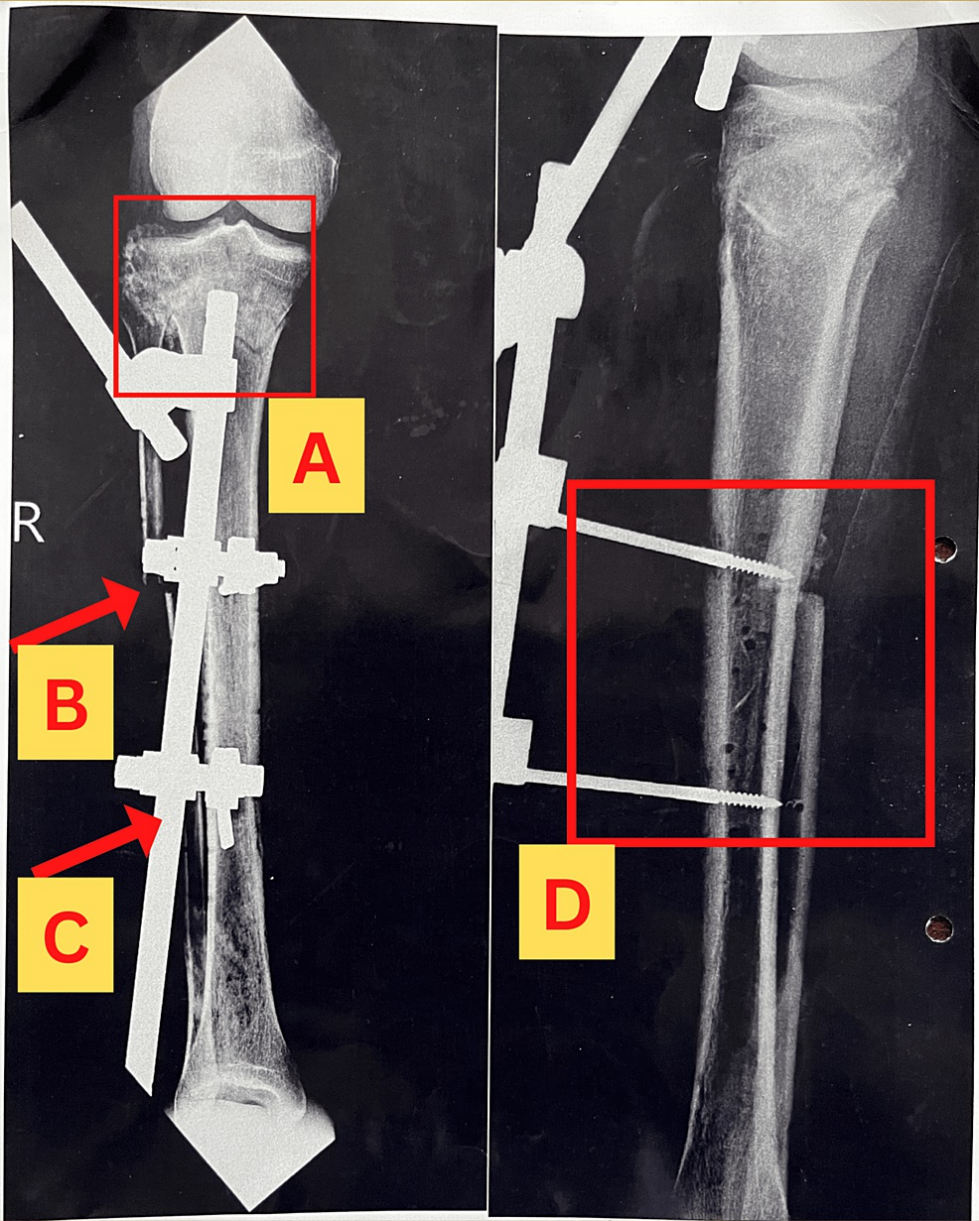
X-ray showing the anteroposterior view (left) and lateral view (right) of the right leg obtained after admission to the orthopedic ward. The square marked as "A" shows the healing proximal tibial fracture associated with the fibular fracture pointed by the arrow marked as "B." Non-bridging external fixator is pointed by arrow "C." The left side of the image represents a radiograph of the anteroposterior view of the leg in which the square marked as "A" shows the healing proximal tibial fracture associated with the fibular fracture pointed out by the arrow "B", whereas the non-bridging external fixator is pointed by the arrow. The right side of the image represents the lateral view of the leg showing external fixation sites along with bony erosions of the tibia sowed in the square marked as "D."

**Figure 7:**
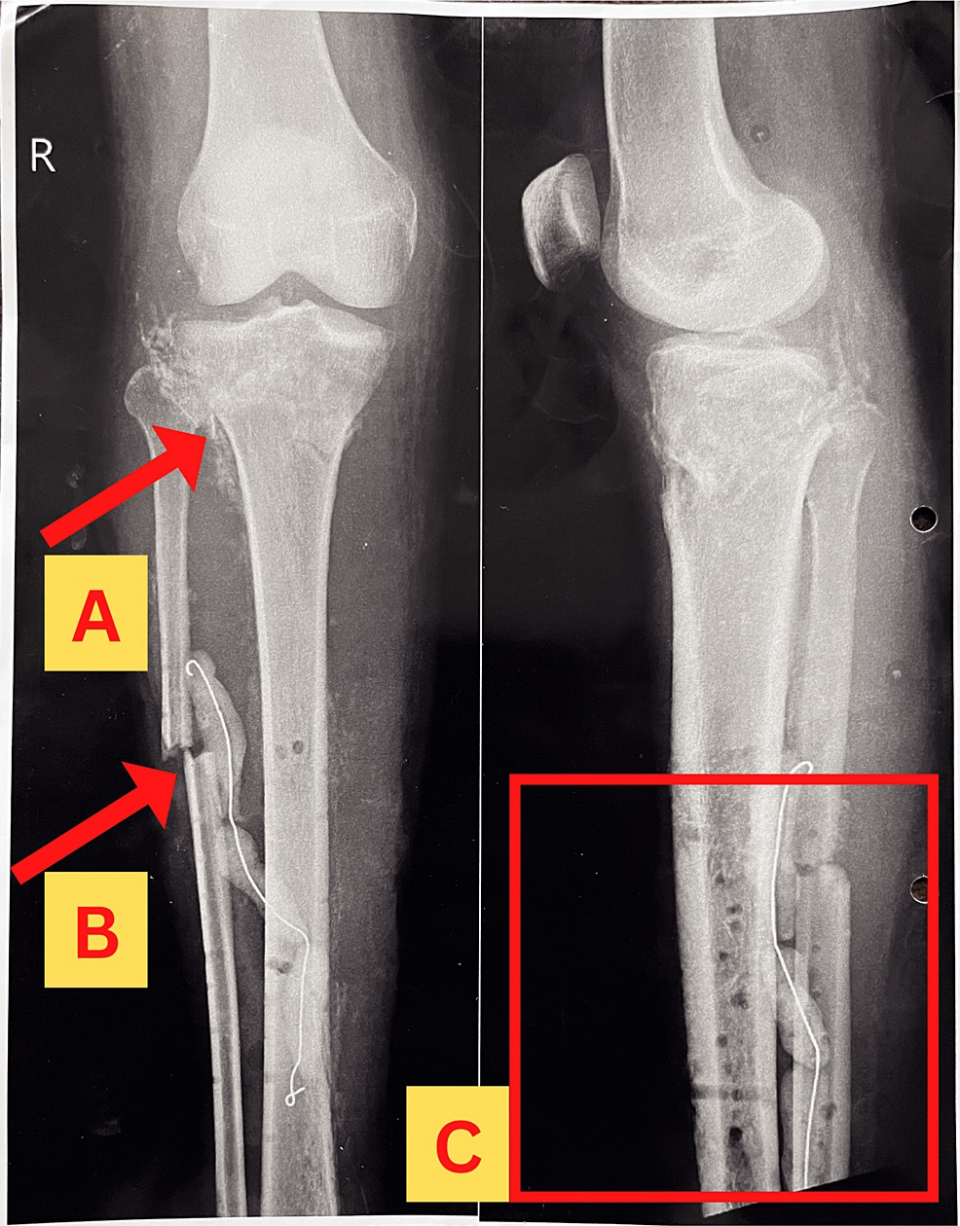
X-ray showing the anteroposterior view (left) and lateral view (right) of the right leg obtained after the removal of the non-bridging external fixator. The left side of the image represents the X-ray of an anteroposterior view of the leg in which an arrow marked as "A" points to the healing proximal tibial fracture, and arrow "B" represents the fibular fracture. The right side of the image represents the X-ray of a lateral view of the leg in which the square marked as "C" shows the erosive changes of the tibial shaft.

**Figure 8:**
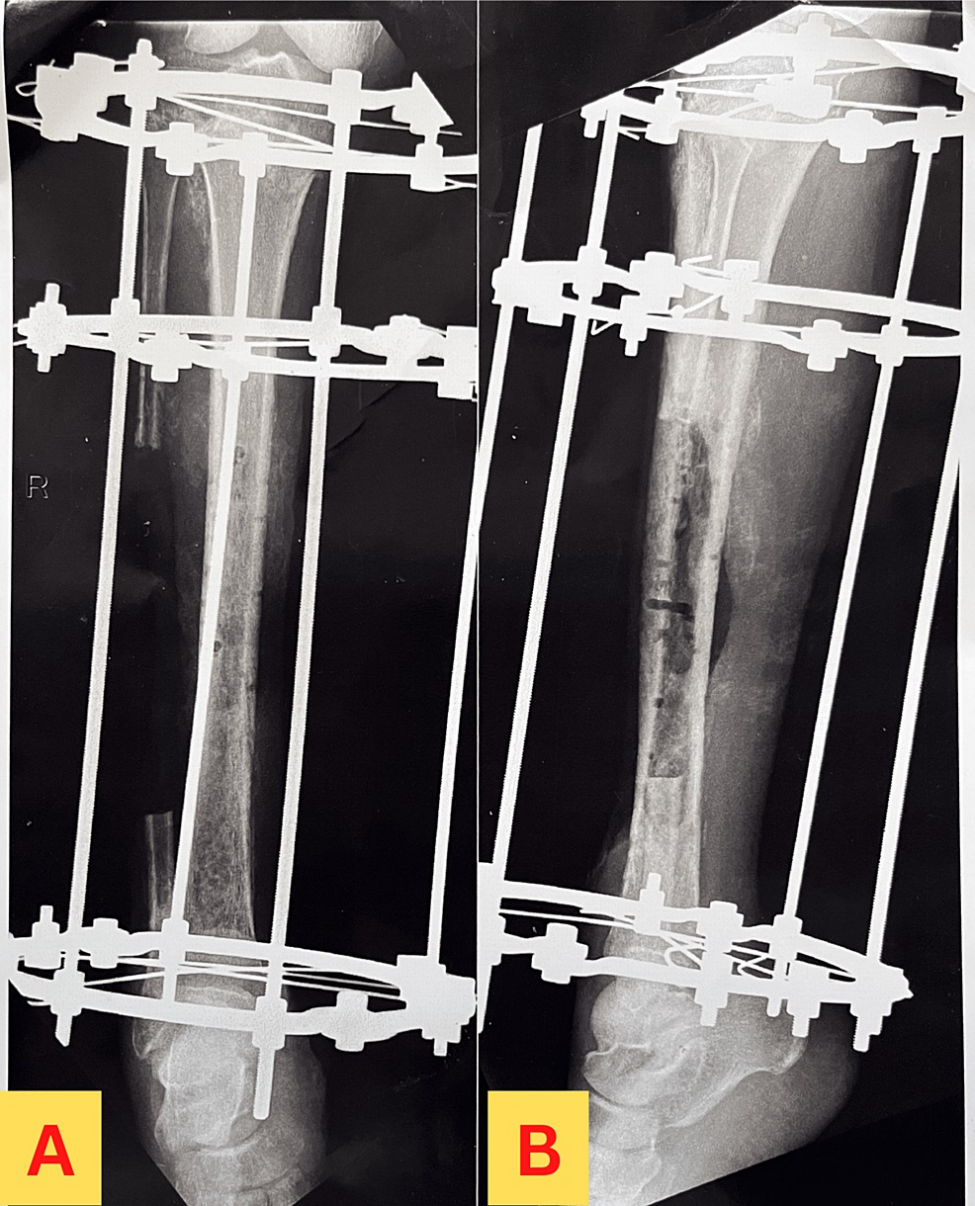
X-ray showing the anteroposterior view and lateral view of the right leg after the application of the Ilizarov fixator. Image A represents the X-ray of the anteroposterior view of the right leg after the application of the Ilizarov fixator. Image B represents the X-ray of the lateral view of the right leg after the application of the Ilizarov fixator.

Radiographs of the affected extremity were obtained before admission to the orthopedic ward, which revealed the findings as shown in Figure 6. Another radiograph was obtained after the first operation, which consisted of debridement of the wound and removal of external fixation from the prior surgery. The findings are shown in Figure 7. The patient underwent another operation, which included fixation with an Ilizarov ring fixator, following which a radiograph was obtained, which revealed findings as shown in Figure 8.

Physiotherapy interventions

**Figure 9:**
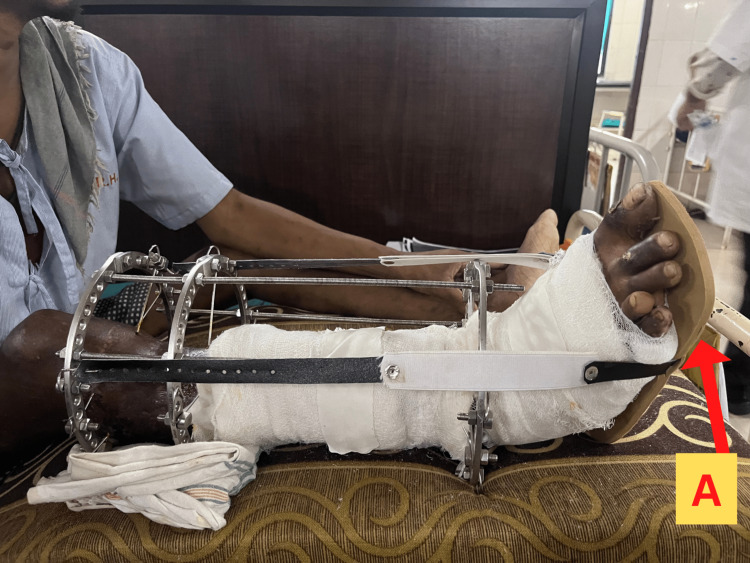
Image showing the right leg with Ilizarov fixator and application of foot drop splint. The arrow "A" points at the foot drop splint applied to keep the ankle and foot in the neutral position.

**Figure 10:**
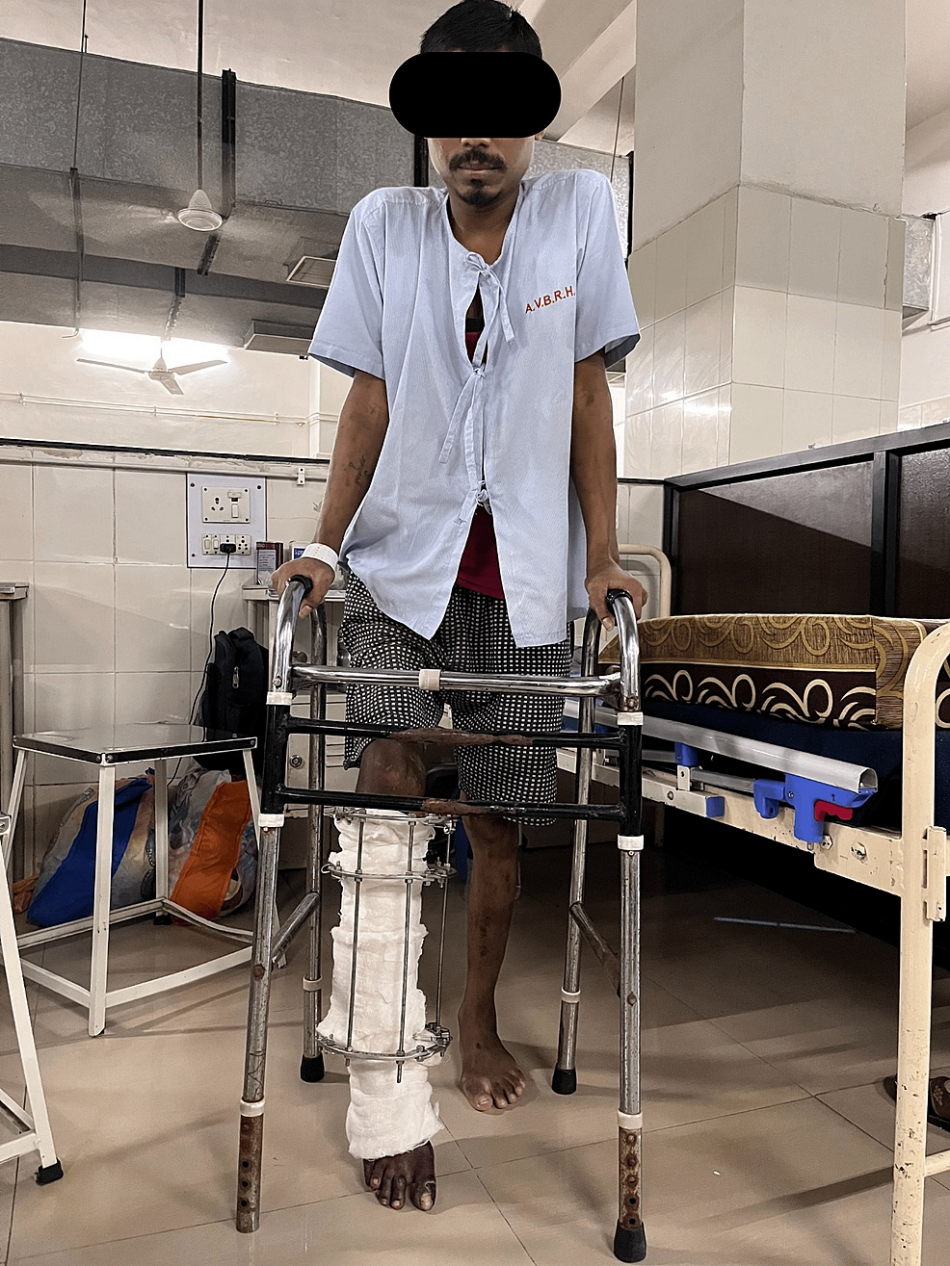
Partial weight-bearing and ambulation training with the use of a walker was executed.

The goal of rehabilitation would be to facilitate the patient to attain an optimal level of independence in performing activities of daily living (ADLs) and functional activities and, thereby, improve his quality of life (QOL). We encouraged the application of a foot drop splint over the right foot to keep it in a neutral position, as shown in Figure 9. We employed sensory re-education training from the first week itself to enhance the sensory function of the right foot. Non-weight-bearing standing and ambulation with the walker were initiated in the second week and then progressed to partial weight-bearing standing and ambulation with the walker in the third week, as shown in Figure 10. Table 2 gives the summary of the week-wise physiotherapy protocol for the given case.

**Table 2:** Summary of the structured week-wise protocol of the early-stage physiotherapy interventions applied in the given case. ACBT: active cycle of breathing technique; ROM: range of motion.

Week	Goals	Interventions	Frequency	
1^st^ week	To prevent postoperative complications like chest infection, deep vein thrombosis, pressure sores, and deformities	Proper positioning of the patient and cushioning of the prominent sites for pressure sores.	Daily	
		Regular change of position and teach pressure relieving maneuvers.		
	Reduce pain and inflammation			
		Use of a foot drop splint to keep the ankle in the neutral position, as shown in Figure 9.		
	Improve range of motion			
	Improve muscle strength	Breathing exercises: ACBT, pursed lip breathing, teaching effective coughing techniques, and incentive spirometry.		
	Improve sensations and motor functions in the right foot			
		Active ankle-toe movements for the left lower limb and passive ankle-toe movements for the right lower limb due to motor impairment.		
	Improve functional independence			
	Cryotherapy for 5 minutes.			
	Quadriceps femoris, hamstrings, and glutei isometric strengthening ­­­- 1 set of 10 rapid contractions on the first day and then progressed to 1 set of 10 repetitions with 10 seconds holds each.		
	Passive straight leg raises (1 set of 10 repetitions).		
	Unilateral bridging (weight bearing on the left lower limb) - 1 set of 10 repetitions.		
	The sensory re-education technique was applied using multiple sensory stimuli of different textures and characteristics. Stroking of vibratory stimuli over the dorsum and plantar aspect of the right foot in proximal to distal direction.		
	Application of galvanic stimulation to the dorsiflexor group of muscles.		
	Dynamic strengthening exercises for unaffected left lower limb and bilateral upper limbs - 1 set of 10 repetitions)		
	2^nd^ week	Reduce pain		Cryotherapy for 5 minutes.	Daily	
Improve ROM		Quadriceps femoris, hamstrings, and glutei isometric strengthening - 2 sets of 10 repetitions with 10 seconds hold each.			
Improve muscle strength					
Improve sensations and motor functions on the right foot					
Improve functional independence					
Passive straight leg raises - 1 set of 10 repetitions.					
Active to active-assisted ROM exercise for affected knee and hip between 0 to 30 degrees that is, heel slides - 1 set of 10 repetitions.					
Unilateral bridging (weight bearing on the left lower limb) - 1 set of 10 repetitions with 5 seconds hold.					
Non-weight-bearing standing and ambulation with the walker.					
The sensory re-education technique was applied using multiple sensory stimuli of different textures and characteristics. Stroking of vibratory and tactile stimuli (moving and static) over the dorsum and plantar aspect of the right foot in proximal to distal direction.					
Application of galvanic stimulation to the dorsiflexor group of muscles.					
Dynamic strengthening exercises for unaffected left lower limbs and bilateral upper limbs.					
3^rd^ week		Improve ROM	Quadriceps femoris, hamstrings, and glutei isometric strengthening - 3 sets of 10 repetitions with 10 seconds hold each.	Daily		
		Improve muscle strength				
	Improve sensations and motor functions in the right foot			
	To improve functional independence	Vastus medialis oblique strengthening exercise 1 set of 10 repetitions.		
	Active assisted straight leg raises - 1 set of 10 repetitions.			
	Active ROM exercises for the right knee and hip joints between 0 and 45 degrees - 1 set of 10 repetitions.			
	Unilateral bridging with weight bearing on the left lower limb - 1 set of 10 repetitions with 10 seconds hold each.			
	Partial weight-bearing standing and ambulation with the walker, as shown in Figure 10.			
	The sensory re-education technique was applied using multiple sensory stimuli of different textures and characteristics. Stroking of vibratory and tactile stimuli (moving and static) over the dorsum and plantar aspect of the right foot in proximal to distal direction.			
	Application of faradic stimulation to the dorsiflexor group of muscles.			
	4^th^ week	Improve ROM	Quadriceps femoris, hamstrings, and glutei dynamic strengthening - 1 set of 10 repetitions with 10 seconds hold each.		Daily	
Improve muscle strength					
		Vastus medialis oblique strengthening exercise - 1 set of 10 repetitions with 10 seconds hold each.			
Improve sensations and motor functions in the right foot					
To improve functional independence					
Active straight leg raises - 1 set of 10 repetitions.					
Active ROM exercise for the right knee and hip joints between 0 and 60 degrees - 1 set of 10 repetitions.					
Full weight-bearing standing and ambulation and ambulation with a walker.					
The sensory re-education technique was applied using multiple sensory stimuli of different textures and characteristics. Stroking of vibratory and tactile stimuli (moving and static) over the dorsum and plantar aspect of the right foot in proximal to distal direction.					
Application of faradic stimulation to the dorsiflexor group of muscles.					

Outcomes measures and follow-up

We took follow-up of the patient after four weeks of rehabilitation. We considered the numerical pain rating scale, ROM, resisted isometric muscle testing, lower extremity function scale, and Semmes-Weinstein monofilaments testing as outcome measures. Then, we compared pre- and post-interventional scores as given in Table 3.

**Table 3:** The scoring of pre- and post-interventional outcome measures was obtained following the four weeks of early rehabilitation. The post-interventional scores for each of the outcome measures seem to be much higher than those of the pre-interventional scores following the four weeks of rehabilitation, which indicates significant recovery in the patient in terms of the range of motion, muscle strength, lower extremity function, and sensations.

Outcome measures	Pre-interventional score	Post-interventional score
Numerical pain rating scale	8/10	3/10
Range of motion		
Right hip flexion	10 degrees	55 degrees
Right Knee flexion	10 degrees	60 degrees
Muscle strength (resisted isometric muscle testing)		
Quadriceps femoris	Weak and painful isometric contractions.	Strong and painless isometric contractions.
Hamstrings	Weak and painful isometric contractions.	Strong and painless isometric contractions.
Gluteal muscles	Weak and painless isometric contractions.	Strong and painless isometric contractions.
Lower extremity functional scale	12.5 %	30 %
Semmes-Weinstein monofilaments testing scores for the right foot (graded from 0 to 6).	Grade 0 (no sensations)	Grade 3 (residual protective sensory function)

## Discussion

Mudey et al. [12] discovered in their case report that the functional independence scores increased from 15% to 63.75% with the use of physiotherapy treatment postoperatively on an adolescent girl who had undergone a sequestrectomy procedure for chronic osteomyelitis of the tibia. Mundada et al. [13] concluded that a multi-disciplinary rehabilitation that consists of a specific surgical procedure and individualized physical therapy management protocol for CO managed with sequestrectomy and fixation with an Ilizarov ring results in improved functional ability and, thus, plays a vital role in quick and effective rehabilitation. Our study presented a case of a patient with a 1.5-month-old extra-articular proximal tibial fracture with acute compartment syndrome associated with a neurovascular deficit in the foot. The patient underwent an operation consisting of fasciotomy and external fixation with a non-bridging external fixator. The chronic osteomyelitis of the operated tibia occurred as a complication of the previous procedure. Later, the non-bridging fixator was replaced with the Ilizarov ring fixator. We started physical therapy management on postoperative day three. The complexity of the case required detailed analysis and prompt postoperative care to prevent and treat complications associated with immobility. Since physiotherapy plays a crucial role in the postoperative rehabilitation of patients to hasten their recovery by early mobilization and promote early discharge, it should be encouraged from the early postoperative stage.

Elsoe et al. [14] performed a study to discover the relationships between complex fractures of the tibia and socioeconomic groups, health-related quality of life, and employment. They ascertained that a year after the removal of fixation, the patients reported significantly worse QOL and complex tibial fractures are associated with lower socioeconomic classes and that 27% of patients who had jobs before the injury returned to work at approximately 19 months after the fracture. In our case, the patient is a truck driver and is the only financial source for his family. It poses a challenge to promote early discharge and achieve optimal levels of functional independence, considering the complexity of the case, particularly foot drop deformity and loss of motor and sensory functions of the affected foot. Sensory re-education was initiated from the first day using a multi-sensory approach to re-establish the sensations in the foot as soon as possible.

Sahtarker et al. [15] concluded that fixation with the Ilizarov ring fixator allows management without the risk of epiphyseal damage. The durability of the Ilizarov ring fixator enables early weight bearing without compromising the mobility of the adjacent joints, and as such, early weight bearing prevents muscle wasting and atrophy, and joint stiffness; and healing with minimal complications after the Ilizarov fixation occurs relatively faster than other methods of operation. We delayed the initiation of weight bearing to the third week due to a 1.5-month-old proximal tibial fracture, which was still healing along with the active osteomyelitis in the tibial bone leading to the instability. We were concerned that early weight bearing might interfere with the stability achieved with the Ilizarov ring fixator. Instead, we maintained the strength of the muscles and integrity of the joints with static strengthening and ROM exercises in the first two weeks of rehabilitation. This was in correspondence with the study by Pires et al. [16], which discussed that only static strengthening and open-chain exercises are appropriate during the acute stage of rehabilitation. These exercises are crucial for preventing the wasting of the muscles, which is common following a prolonged period of immobility. As a result, it is critical to incorporate close-chain exercises and concentric and eccentric strengthening exercises that resemble the patient's ADLs. Maintaining optimum ROM of unaffected joints and preventing atrophy of surrounding musculature is critical at all stages.

York et al. [17] concluded in their study that foot drop can occur due to a variety of etiological factors and, thus, the appropriate treatment approaches with optimal outcomes should be individualized for each patient based on the etiology. Combinations of physiotherapy, surgical alternatives, ankle-foot orthosis (AFO), or functional electrical stimulation allow physicians to impart to patients the interventions that meet their needs. However, all the management options available currently emphasize symptomatic care with optimal motor function recovery. In our case, the use of functional electrical stimulation was limited by the presence of Ilizarov fixation, wherein selective stimulation of each involved muscle could not be done. Application of AFO was restricted due to the presence of a slab, thus, a foot drop splint was used to keep the foot in a neutral position.

## Conclusions

The comparison of pre- and post-physiotherapy intervention outcome measure scores after four weeks of rehabilitation suggests significant improvements in ROM, muscle strength, sensory functions, and lower limb functions, and further recovery is expected with the continuation of an individually structured exercise regime. Thus, it can be concluded that the application of physical therapy in the early postoperative rehabilitation stage plays an important role in preventing complications associated with immobility, improving ROM, muscle strength, and overall functional ability of the patient, and thereby facilitating an early return to normal life. The above case report provides a comprehensively structured early-stage physiotherapy management protocol for the patient with chronic osteomyelitis, with the neurovascular deficit and foot drop deformity operatively managed with Ilizarov fixation.
